# Analysis of Pulmonary Vein Antrums Motion with Cardiac Contraction Using Dual-Source Computed Tomography

**DOI:** 10.7759/cureus.712

**Published:** 2016-07-26

**Authors:** Houda Bahig, Jacques de Guise, Toni Vu, Carl Chartrand-Lefebvre, Danis Blais, Martin Lebeau, Nhu-Tram Nguyen, David Roberge

**Affiliations:** 1 Department of Radiation Oncology, Centre hospitalier de l'université de Montréal (CHUM); 2 Medical Imaging, Centre hospitalier de l'université de Montréal (CHUM); 3 Department of Radiology, Centre Hospitalier de l'Université de Montréal (CHUM); 4 Department of Radiation Oncology, Centre hospitalier de l'université de Montréal (CHUM) - Hôpital Notre-Dame; 5 Department of Radiation Oncology, McMaster University-Juravinski Cancer Centre, Hamilton, ON; 6 Department of Oncology, Division of Radiation Oncology, McGill University Health Center; 7 Department of Radiology, Radiation Oncology and Nuclear Medicine, University of Montreal

**Keywords:** radio-surgery, cardiac, atrial fibrillation, dual source ct, pulmonary vein isolation

## Abstract

Purpose: The purpose of the study was to determine the extent of displacement of the pulmonary vein antrums resulting from the intrinsic motion of the heart using 4D cardiac dual-source computed tomography (DSCT).

Methods: Ten consecutive female patients were enrolled in this prospective planning study. In breath-hold, a contrast-injected cardiac 4-dimensional (4D) computed tomography (CT) synchronized to the electrocardiogram was obtained using a prospective sequential acquisition method including the extreme phases of systole and diastole. Right and left atrial fibrillation target volumes (CTVR and CTVL) were defined, with each target volume containing the antral regions of the superior and inferior pulmonary veins. Four points of interest were used as surrogates for the right superior and inferior pulmonary vein antrum (RSPVA and RIPVA) and the left superior and inferior pulmonary vein antrum (LSPVA and LIPVA). On our 4D post-processing workstation (MIM Maestro™, MIM Software Inc.), maximum displacement of each point of interest from diastole to systole was measured in the mediolateral (ML), anteroposterior (AP), and superoinferior (SI) directions.

Results: Median age of the enrolled patients was 60 years (range, 56-71 years). Within the CTVR, the mean displacements of the superior and inferior surrogates were 3 mm vs. 1 mm (p=0.002), 2 mm vs. 0 mm (p= 0.001), and 3 mm vs. 0 mm (p=0.00001), in the ML, AP, and SI directions, respectively. On the left, mean absolute displacements of the LSPVA vs. LIPVA were similar at 4 mm vs. 1 mm (p=0.0008), 2 mm vs. 0 mm (p= 0.001), and 3 mm vs. 1 mm (p=0.00001) in the ML, AP, and SI directions.

Conclusion: When isolated from breathing, cardiac contraction is associated with minimal inferior pulmonary veins motion and modest (1-6 mm) motion of the superior veins. Target deformation was thus of a magnitude similar or greater than target motion, limiting the potential gains of cardiac tracking. Optimal strategies for cardiac radiosurgery should thus either incorporate the generation of an internal target or cardiac gating. In either case, cardiac 4D DSCT would allow for personalized margin definition.

## Introduction

Atrial fibrillation (AF) is the most common type of cardiac arrhythmia, with an estimated prevalence of four million in the United States [1], and up to 70% of the cases occurring in patients aged between 65 and 85 years old [2]. Its consequences involve increased risk of death, increased thromboembolic events as well as decreased quality of life [1]. AF is caused by aberrant electrical impulses from the pulmonary veins entering the left atrium, causing ineffective rapid contraction of the left atrium leading to an irregular heart rhythm and potential thrombus formation [3]. Electrical isolation of the pulmonary veins through catheter ablation is a well-established treatment approach for atrial fibrillation that has been associated with up to 60% control of AF at five years [4]. Pulmonary vein isolation by catheter ablation is an invasive procedure that is associated with up to 6% complications rate, including thromboembolic events, myocardial infarction, cardiac tamponade, oesophageal injury, or even death [5-6]. A significant proportion of the elderly AF population (with frequent co-morbidities) are thus ineligible for the procedure.

Stereotactic radiosurgery (SRS) as a treatment for AF has been investigated in a limited number of studies. In fact, the ability to create a fibrotic cardiac lesion [7-10] and the potential to induce an electrophysiological effect have been suggested in animal studies [7, 11]. SRS potential resides in offering a non-invasive alternative to older patients with significant comorbidities. When considering cardiac SRS, respiratory motion and cardiac contraction both contribute to target motion. Whereas several techniques such as breath hold, respiratory gating, or near real-time tracking are used to take into account respiratory motion, cardiac contractions remain challenging. Respiratory tracking has been investigated using the Cyberknife system (Accuray Inc. Sunnyvale, USA) with placement of fiducial markers near the target volume [7-9] as well as, more recently, using real-time cardiac magnetic resonance imaging [12]. Ipsen et al., [12] conducted a dosimetric study in one human subject targeting all four pulmonary veins antrums. They showed that with the use of larger safety margins, heart and esophagus dose constraints were exceeded, and target volume coverage was compromised, therefore emphasizing the importance of minimizing unnecessary margins. Whether patients are treated under respiratory tracking method or using a breath-hold technique, a better understanding of the dynamics of cardiac displacement across the four pulmonary vein antrums is needed. In this study, we used a dual-source computed tomography (DSCT) synchronized to the patient’s electrocardiogram in order to assess diastole-to-systole associated cardiac displacement [13]. The purpose of the study was to determine the extent of displacement of each pulmonary vein antrum resulting from cardiac contraction in order to generate individualized internal target volume margin accounting for cardiac deformation.

## Materials and methods

### Study population

Ten consecutive female patients with left breast cancer aged 18 years and older and no known allergy to iodine contrast were prospectively enrolled in a study of cardiac DSCT radiotherapy simulation between July 2015 and March 2016. All patients had a baseline electrocardiogram and biochemistry including creatinine level. The protocol and patient consent form were reviewed and approved by our institutional ethics committee.

### Cardiac 4D DSCT

A contrast-injected (Isovue 370), cardiac 4D DSCT synchronized to the patients' electrocardiogram (ECG) scan was acquired on a Somatotom Flash Definition (Siemens Healthcare, Erlangen, Germany). Contrast injection was at a rate of 4ml/sec and had three phases: a) 60 ml of contrast over the first fifteen seconds, b) 10 ml of contrast and 10 ml of Sodium Chloride (NaCl) 0.9% over the following five seconds, and c) 40 ml of NaCl 0.9% over the final ten seconds. With a rotation time of 0.28 second using a detector with 64 × 0.6 mm beam collimation, the acquisition time for the entire cardiac volume was <0.3 seconds at a temporal resolution of 75 milliseconds [13]. The cardiac 4D DSCT was obtained in the supine position with arms up, using a breast board and vaclock as immobilization devices.  A prospective sequential acquisition method including eight phases of the cardiac cycle (20%, 30%, 40%, 50%, 60%, 70%, 80% and 90%) including the extreme phases of systole and diastole was used. Cardiac 4D DSCT was acquired in breath-hold (either deep inspiration breath-hold or natural inspiration breath-hold reproduced using an Abches system (APEX Medical, Tokyo, Japan)). ​

### Pulmonary vein motion assessment

Two clinical target volumes (CTV) were defined for isolation of all four pulmonary veins [12]: a right CTV (CTVR) which included the right superior and inferior pulmonary vein antrums (RSPVA and RIPVA) and a left CTV (CTVL) which included the left superior and inferior pulmonary vein antrums (LSPVA and LIPVA). Four regions of interest (ROI) were defined as a 5 mm surface on axial view at the most antero-superior intersection of each pulmonary vein with the left atrium. These ROIs representing the RSPVA, RIPVA, LSPVA, and LIPVA were contoured on each of the eight cardiac phases by a same observer with particular care to ensure ROI was segmented at the same location in all phases. Maximal displacement of the centroid of each ROI was derived automatically in a MIM Maestro workstation (MIM Software Inc., Cleveland, OH) in the mediolateral (ML), anteroposterior (AP), and superoinferior (SI) directions.

Data were collected in an encrypted electronic database. Student T-test was used to compare maximal displacements. Analyses were completed using the SPSS statistics package (IBM Corp, 2013. IBM SPSS Statistics for Windows, Version 22.0. Armonk, NY). 

## Results

### Patients characteristics

Ten female patients with a median age of 60 years (range, 56-71 years) were included in this study. Baseline ECG showed sinus rhythm in all patients, and none of the patients had a past medical history of AF, congestive heart failure, or valvular disease. One patient (patient #8), was on an angiotensin II receptor antagonist for hypertension. No patient presented anatomical variants such as a common pulmonary trunk or an accessory vein. All patients presented a total of four pulmonary veins. Table 1 summarizes basic scan parameters.

**Table 1:** Basic DSCT-Related Parameters CTDIvol= volume CT dose index, DLP= dose length product, mSv= milisievert, N= number of patients, DIBH= deep inspiration breath-hold. Effective dose was estimated by the product of the DLP and a conversion coefficient for the chest (k= 0.014 mSv*mGy-1*cm-1).

Scan-Related Parameters	Mean	Range
CTDIvol-32 cm (mGy)	60.7	42.3-73.0
DLP (mGy-cm)	1072	729-1554
Effective dose (mSv)	15	(10-22)
	N	%
DIBH	4	40%

### Motion analysis

**Figure 1:**
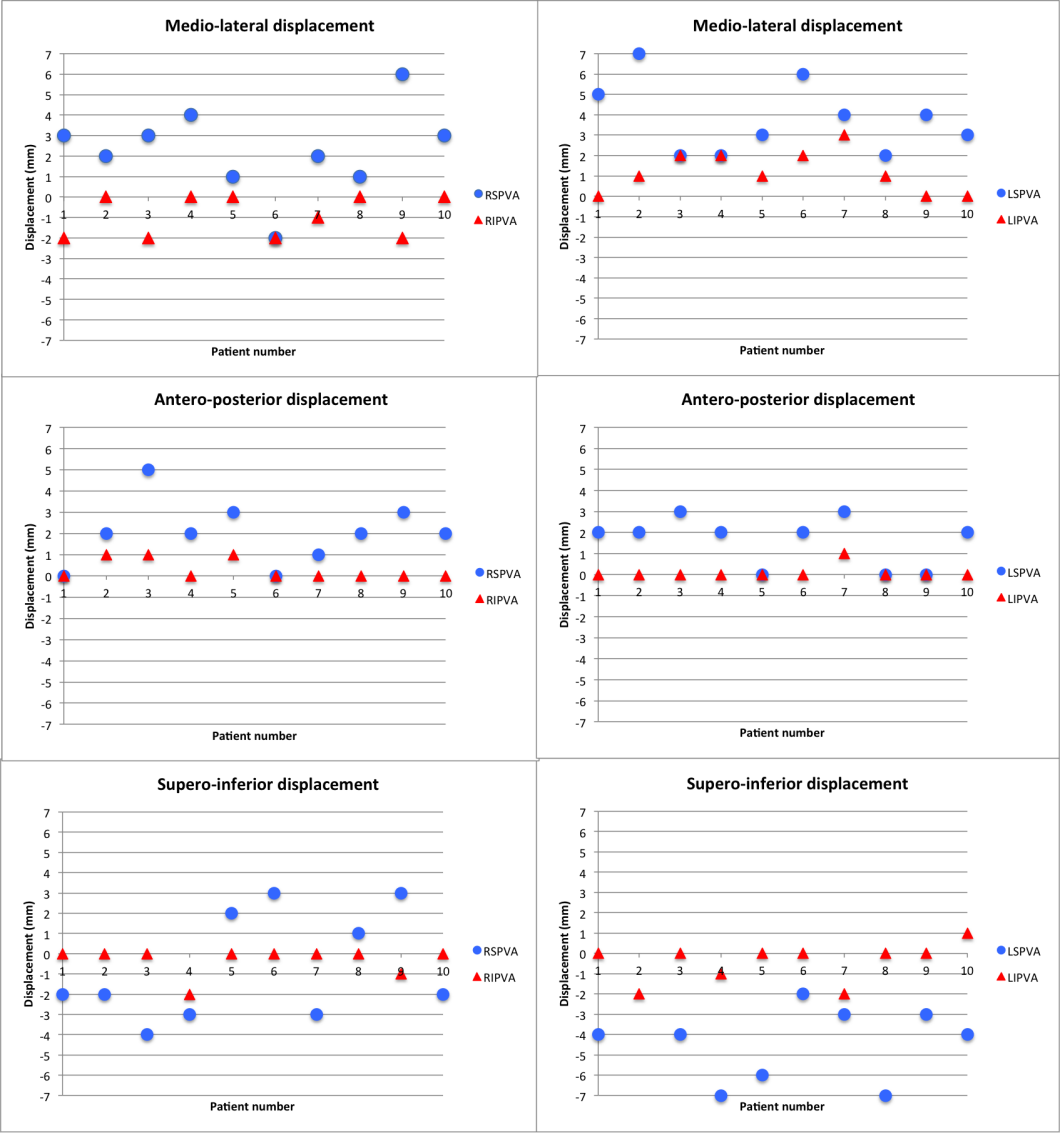
Plots of Maximum Cardiac Displacement of Right Superior vs. Right Inferior Pulmonary Antrums (A), as well as Left Superior vs. Left Inferior Pulmonary Vein Antrums (B) RSPVA= right superior pulmonary vein antrum, RIPVA= right inferior pulmonary vein antrum, LSPVA= left superior pulmonary vein antrum, LIPVA= left inferior pulmonary vein antrum.

Plots of maximum cardiac displacement of right superior vs. right inferior pulmonary antrums as well as left superior vs. left inferior pulmonary vein antrums are shown in Figures 1A-1B, respectively.

**Figure 2:**
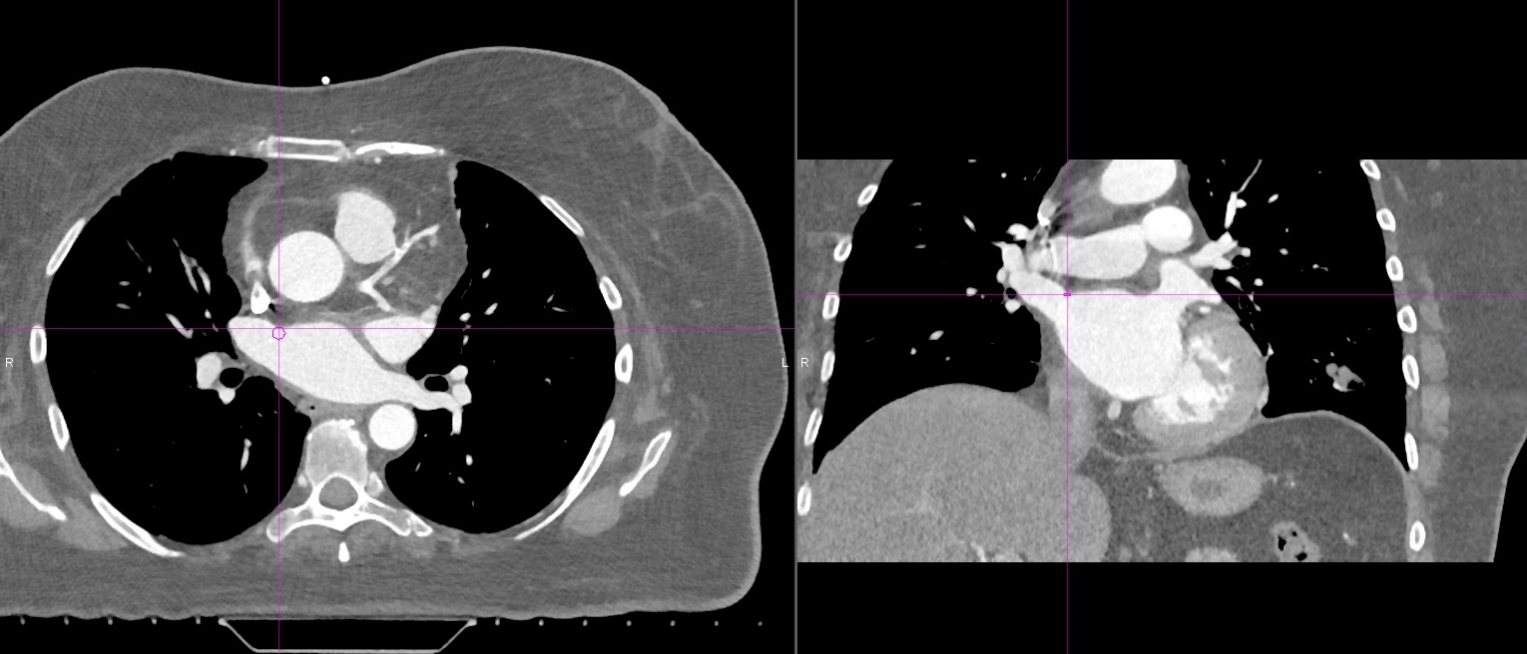
ROI Representing the RSPVA Defined as a 5 mm Axial Surface at the Anterosuperior Intersection of the Superior Pulmonary Vein with the Left Atrium ROI= region of interest, RSPVA= right superior pulmonary vein antrum

Displacements shown represent the motion of each structure in the ML, AP, and SI directions, from diastole to systole, secondary to deformation of the CTVR and CTVL. These plots show the that magnitude as well as the direction of the displacements are different for the superior and inferior veins. Figure 2 shows a 5mm ROI in the axial plane at the most anterior intersection of the right superior pulmonary vein with the left atrium, representative of the RSPVA. 

Mean displacements of RSPVA, RIPVA, LSPVA, and LIPVA are shown in Table 2.  For the CTVR, mean absolute displacement of RSPVA vs. RIPVA was 3 ±2 mm vs. 1 ±1 mm (p=0.002), 2 ±1 mm vs. 0 ±0 mm (p= 0.001), and 3 ±3 mm vs. 0 ±1 mm (p=0.00001), in the ML, AP, and SI directions, respectively. For the CTVL, mean absolute displacement of LSPVA vs. LIPVA was 4 ±2 mm vs. 1 ±1 mm (p=0.0008), 2 ±1 mm vs. 0 ±0 mm (p= 0.001), and 3 ±2 mm vs. 1 ±1 mm (p=0.00001) in the ML, AP, and SI directions respectively.

**Table 2:** Mean Displacements of RSPVA, RIPVA, LSPVA, and LIPVA RSPVA= right superior pulmonary vein antrum, RIPVA= right inferior pulmonary vein antrum, LSPVA= left superior pulmonary vein antrum, LIPVA= left superior pulmonary vein antrum. SD= standard deviation, ML= mediolateral, AP= anteroposterior, SI= superoinferior.

	ML (mm)			AP (mm)			SI (mm)		
	Mean	SD	p	Mean	SD	p	Mean	SD	p
RSPVA	3	± 2	0.002	2	± 1	0.001	3	± 3	0.00001
RIPVA	1	± 1		0	± 0		0	± 1	
LSPVA	4	± 2	0.0008	2	± 1	0.001	3	± 2	0.00001
LIPVA	1	± 1		0	± 0		1	± 1	

## Discussion

In this study, we determined the extent of displacement of the pulmonary veins resulting from the contractile motion of the heart. Using a cardiac 4D DSCT, we have shown that margins accounting for cardiac deformation can be individualized and that inferior pulmonary veins were significantly less mobile than their superior counterpart. We found that mean displacement of the superior pulmonary veins was typically 4 mm in the ML direction (reaching up to 8 mm in one patient), and 3 mm in the SI direction (reaching up to 7 mm in one patient). Mean displacement of the inferior veins was 1 mm or less in all directions. Our study was conducted in healthy individuals with no known AF. Cardiac anatomy remodeling in patients with AF has been described [14], and therefore, it is possible that our reported pulmonary veins motion dynamics be different from that expected in patients with known AF. In addition, our results may be limited by the uncertainty related to intra-observer variability in the measure of pulmonary veins displacements. However, displacements reported in our study are similar to the results from a study by Rettman et al. [15]. In that study, clips were placed within the pulmonary vein ostia and left atrial appendage of canine hearts; average displacement of eleven clips placed in three canine hearts was 2 mm, 2 mm, and 1 mm in the ML, AP, and SI directions, respectively. Importantly, the definition of a precise target volume for cardiac radiosurgery is poorly defined in the literature. In addition, no patient included in our study had a known diagnosis AF, which may limit the applicability of our findings to healthy individuals. AF is particularly challenging when it comes to cardiac imaging, as the rapid rates are associated with significant motion artifacts and impaired image quality. A distinctive feature of our study is the use of DSCT which provides high temporal resolution even in patients with AF rhythm [16].

Literature on the role of radiosurgery for the treatment of AF by pulmonary vein isolation remains preliminary. A study on mini swine using the Cyberheart system (Portola Valley, CA), where fiducials were implanted next to the target volume, demonstrated the feasibility of using stereotactic robotic radiosurgery to create cardiac fibrotic lesions as well as to induce a significant decrease in voltage at the pulmonary vein–left atrial junction at a dose of 25 Gy [7]. In another animal study targeting the right pulmonary vein ostia, the use of in-vivo thermoluminescent dosimeter near the right pulmonary vein showed that the accuracy of the CyberKnife radiosurgery system within 5% of the predicted dose [9]. Using an internal target volume method accounting for both respiratory and cardiac motion, Bode et al., [11] investigated the feasibility of lesion formation in a porcine model. Radiation doses between 23 and 40 Gy were delivered to the right superior pulmonary vein, to which additional margins of 2-3 mm and 10-15 mm for cardiac and respiratory motion were added, respectively. At six months, right pulmonary vein voltage was reduced, and pathological analysis revealed transmural scarring with doses beyond 30 Gy [11]. However, reported toxicities included broncho-mediastinal fistula and AV node block [11]. Importantly, treatment of all four pulmonary veins would have been associated with a significantly higher treatment volume and likely increased toxicity, highlighting the importance of minimizing internal target volume margins when possible. Using a target volume similar to our study, Ipsen et al., [12] conducted a planning study on one patient with AF where all four pulmonary vein antrums were targeted. Using an incremental safety margin from 0 to 8 mm, the authors reported that increasing the margins was associated with exceeding of heart and esophagus normal tissue tolerance and significant compromise on the target volume coverage.

The heart is subject to two sources of motion during treatment: respiratory motion as well as intrinsic cardiac contraction. If respiratory motion management is now commonplace through breath hold, tracking, or gating; cardiac contraction remains a challenge. Delivering a dose that can create fibrosis in the pulmonary vein antrums without complications to the surrounding healthy tissues requires maintaining high spatial dose gradients. In our study, we showed that cardiac contraction is associated with deformation of target structures, leading to different displacements of the superior and inferior pulmonary veins, that cannot be taken into account by simple tracking. Whether the patient respiratory motion is taken into by the Cyberknife System, real-time MRI, or with breath-hold technique, individualizing assessment of cardiac motion would allow for selection of optimal margins for deformation. An alternative strategy would reside in the development of a cardiac-gated treatment synchronized to ECG signal [17], in which case the multiphase DSCT would help in choosing an optimal gating window.

## Conclusions

We found cardiac contraction to be associated with negligible inferior pulmonary vein displacement and a mean superior pulmonary vein displacement of 4 mm. This difference is the result of cardiac deformation and highlights the potential limitations of cardiac tracking. In an internal target volume strategy accounting for cardiac deformation, cardiac 4D DSCT would allow for personalized assessment of the displacements of the superior and inferior pulmonary veins and selection of optimal margins allowing optimal target coverage while avoiding unnecessary irradiation of healthy tissues.
